# Developing a national road traffic safety education program in Iran

**DOI:** 10.1186/s12889-020-09142-1

**Published:** 2020-07-06

**Authors:** Fatemeh Bakhtari Aghdam, Homayoun Sadeghi-Bazargani, Saber Azami-Aghdash, Alireza Esmaeili, Haneieh Panahi, Maryam Khazaee-Pool, Mina Golestani

**Affiliations:** 1 Road Traffic Injury Research Center, Tabriz University of Medical Sciences, Tabriz, Iran; 2 Department of Health Education & Promotion, Tabriz University of Medical Sciences, Tabriz, Iran; 3 Tabriz Health Services Management Research Center, Health Management and Safety Promotion Research Institute, Tabriz University of Medical Sciences, Tabriz, Iran; 4 HRM & traffic safety Department, Tehran University of police, Tehran, Iran; 5 Department of Promoting & Rural Development, Master of Science in Agricultural Engineering the tendency for rural development, Tabriz University, Tabriz, Iran; 6 Department of Public Health, School of Public Health, Mazandaran University of Medical Sciences, Sari, Iran; 7 Health Sciences Research Center, Addiction Research Institutes, Mazandaran University of Medical Sciences, Sari, Iran

**Keywords:** Traffic safety, Educational program, Traffic behavior, National program

## Abstract

**Background:**

Education is a pivot included Decade of Action for Road Safety initiative, which has reduced deaths caused by accidents in developed countries. Given the circumstances of each country, a proper education program is necessary. Thus, the aim of this study was to develop the National Road Traffic Safety Education Program (NRTSEP) and reduce Road Traffic Injuries (RTIs).

**Methods:**

This study used a qualitative approach and was conducted in Iran. Data were obtained through sixteen semi-structured in-depth interviews from indifferent road safety and health promotion field experts as well as eleven focus group discussion (FGD) sessions conducted with participants from general population. Inductive qualitative content analysis was used to converge and compare themes through data. The initial pattern of the program was developed and subsequently, the designed program was validated and finalized by two-step Delphi method for the consensus of expert opinion.

**Results:**

The following six main themes emerged from the analysis: target groups, program content, educational methods, instructors, resources and evaluation. The target group consisted of children, youth, parents, teachers, elderly, motorcyclists, cyclists, pedestrians, drivers, driver license applicants, instructors and administrators of driving schools, and specific groups such as the disabled, managers at different levels, and policymakers. The content of the program consisted of 27 items, including traffic laws and regulations, first aid, and medical emergencies. Educational practices and authorities were determined based on the target group and educational content. The most important resources of the program were human force and other cases, which can be managed in case of a lead agency. In the evaluation dimension, the cases such as mortality rate, hospitalization, behavioral changes, and other cases can be considered.

**Conclusions:**

The designed program should be implemented for all target groups for road safety promotion. The proper content was provided with proper educational methods and instructors for the target groups. A lead agency is needed to provide the resources and funding to run the program.

## Background

In Iran, Road Traffic Injuries (RTIs) are the leading cause of injuries and the second leading cause of death [1, 2]. In recent years, like most of the developing countries, various interventions and programs have been designed and implemented to reduce RTIs [3–6]. Some of these interventions and programs, including severe treatment with speeding, violations, improvements to vehicle safety, and raised fines and penalties, have a moderate impact on reducing the rate and burden of accidents and some of these interventions, such as increased police funding, security of roads, speed cameras, improved emergency medical care services, and development of transportation infrastructures, especially for the rail transportation, have a slight impact on reduction of traffic accidents [7].

Basically, in road accidents, three factors of human, road, and vehicle are identified as major components, and the quality and quantity of displacements will be affected by these three factors. Human is the most unknown and most important factor in the course of events and, with all the efforts of experts in various sciences, the human reactions in dealing with the events cannot be predicted. Many differences between human beings, including physical, mental, etc., lead to the unknown nature of humans, such that above 70% of the accidents are attributed to this factor. Although the human factor has its own characteristics in various communities, the human factor, versus other causes, is the most important and difficult part for controlling transportation systems [8]. The analysis of road traffic accidents in Iran suggests that the most common factor involving RTIs could be the human factor. In fact, in traffic crashes in Iran, the human factor alone or along with other factors is often responsible for the majority of traffic accidents [9, 10].

The behavior of pedestrians, motorcyclists, and cyclists is a human factor involved in the occurrence of traffic accidents. Due to the role of human factor in the occurrence of accidents, traffic safety education programs are needed to be implemented. The public needs to become aware that the issues of traffic and traffic accidents are vital to their health and safety. In order to reduce traffic injuries policies such as improving the behavior of road users are implemented in countries such as Australia that are pioneering the reduced RTIs and crash fatalities [11] . In Germany, through traffic education in schools, the mortality rate of 19,000 in 1970s was reduced to 9000 in 1995. In Hamburg, Germany, the deaths caused by accidents became stable around 100 people per year by education in all groups from kindergarten to university and even holding courses for the elderly [12]. Other studied showed that it is necessary to develop road safety education [13, 14].

Different studies in Iran has shown that, despite the implementation of specific laws such as the use of seat belts, the rate of deaths has had small decline without education, demonstrating the need for education and cultural development in the country [5, 15].

Therefore, the role of education in reducing traffic accidents is inevitable and the educational programs for the population must be provided and implemented based on the resources and facilities as well as the environmental, economic, political, and cultural conditions of each society.

Considering the human factors for the occurrence of accidents from 2006 to 2016 in Iran, it is concluded that most of these factors can partially be eliminated through education. The major factors, including violation of traffic laws [10, 16–19] and inadequate information [10, 20], are the other cases, in which education can play an important role for changing behaviors. Therefore, it is essential to pay attention to education and cultural development that lead to behavioral changes [21, 22]. Therefore, given the importance of accidents as the second leading cause of death in Iran and the necessity of education with respect to the facilities, and social, cultural, and political contexts of Iranian society. The aim of the present study was to develop the National Traffic Safety Educational Program (NRTSEP) and reduce Road Traffic Injuries (RTIs).

## Methods

As part of the project for developing the National Iranian Road Safety Program, this qualitative study was conducted to develop a National Traffic Safety Educational Program (NRTSEP) in 2019. Semi-structured in-depth interview and focus group discussions (FGD) were used to collect data. Participants in the needs assessment were recruited from of different setting of Tabriz and the traffic experts from Tehran, Mashhad and Shiraz were interviewed. In order to gain a different viewpoint, a purposive sampling method was applied to insure that participants with varying levels of socioeconomic status, age, educational level, job, personal or family accident records, and driving violations were present in this work. At the beginning of interviews, all of participants were informed about the aim of the study.

### Focus group discussions (FGD)

Overall, eleven FGD were conducted with different groups including mothers with young children; parents with students; the elderly; professional drivers; motorcyclists; cyclists; high school students; university students;, teachers; offending drivers and elementary students. Sessions were performed in convenient places in reach for all the participants. Each session lasted for about 1 h

### Semi-structured interviews

Sixteen semi-structured interviews were conducted with stakeholders and experts on road safety and health promotion in Tabriz¸ Tehran, Mashhad and Shiraz as well as various organizations with the same conditions a Ministry of Roads and Urban Development, professors and experts of Ministry of Health and Medical Education, police experts, Ministry of Education, forensic medicine, Central Insurance Organization, Iranian Red Crescent Society, National Medical Emergency, Law Enforcement Force, and IRIB. In the selection of participants, the heterogeneous method (participants with the most diversity of various specialties) was used. Sampling was continued until data saturation reached, that is, no new codes were identified in the data. The semi-structured interview guide forms were used for the interviews, for which the literature review and opinions of various professors in the fields related to the subject and the research team were used. The interview began with the open-ended questions, such as “Which groups should be taught to reduce traffic accidents?” and continued with these questions “What content should be taught?” and “Who should be taught?”. After each question, participants were invited to explain more about what they had reported. The interviews were performed at the expert office’s or at a convenient public place. Each interview lasted 60–120 min.

### Delphi survey

Based on the results of previous stages (needs assessment of people and experts) and the opinions of the research team, a primary suitable educational program was formulated. Then, selected educational program were entered into the Delphi survey phase.

### Data analysis

Inductive qualitative content analysis (a bottom-up method to explore the data) was employed based on the Graneheim method to converge and compare themes through participant data. Themes were clustered pursuant to participants’ views. In this method, the researcher recognized the themes based on the primary codes and categories. As such, the units of analysis are the entire interviews [23]. Data analysis started throughout the data-gathering process. Each FGD and individual interview was transcribed literally and analyzed before the next FGD or interview was accomplished. Thorough understanding of the data was reached by frequently reading the transcriptions. All transcripts were imported into the MAXQDA software v. 10 which is a method for identifying, analyzing, and reporting themes within the context and is widely used in qualitative data analysis. Data coding was conducted by two of the researchers. Delphi questionnaire was designed using the Likert scale in order to assess the validity and consensus of the formulated educational program and was given to 10 experts as expert pannel. After this step, the educational program was modified and became ready to use. The questionnaire consisted of three parts, including a brief introduction to the goals and necessity of the study, a completion guide for form, and forms.

To analyze Delphi method, each of the individuals scored each dimension and area of the educational program in two perspectives of importance (Does this perspective matter and should it be taken into consideration?) and applicability (Is there applicability for this area of the desired dimension?). In this section, the individuals first chose their general idea from three options of “Disagree”, “No idea” and “Agree” and, then, based on their previous choices, a score from 7 to 4 was given to each role given the previous option. In the information analysis step of this section, the scores were sorted from right to left. For example, the number 4 in “Disagree” was considered as 7 and number 4 in “Agree” was considered as 9. The number assigned to “No idea” was 5 [24].

### Validation

In order to confirm the validity of the program, the content validity index and modified Kappa were used. For validation in Delphi phase was two rounds of Delphi technique. The first round of the Delphi phase was conducted with 20 experts and scholars of traffic accident. Delphi forms were sent to individuals through emails and 2 weeks were given for completion; after this period, a reminder email was sent again. The second round of Delphi was conducted with 10 people. In this phase, 2 weeks were given to individuals to complete the forms. In this step, due to the high agreement of the first and second phases, the Delphi technique was finished and the program was finalized. Delphi questionnaire consisted of 9 points on the Likert scale and areas with scores above 7 were accepted and areas with mean 4–7 entered the second phase; areas with a score less than 4 were eliminated. The Content Validity Index (CVI) and modified KAPPA were assessed based upon expert perspectives. A minimum cumulative modified KAPPA score of 0.79 is expected to be achieved for the content validation.

## Results

Overall, Six major themes emerged from the analysis forming the six dimensions of NRTSEP, including target group, program content, educational methods, instructors, resources, and evaluation. More information on themes and categories are presented in Table 1.

**Table 1:** Mains themes and categories

Mains themes	Categories (n)	Categories (n)
Target groups	• Preschoolers to university students (153) • Parents (149) • Teachers (148) • Cyclists (141) • Motorcyclists (126) • Drivers (124) • Pedestrians (122)	• Youth (119) • Elderly (118) • Driving license applicants, instructors and managers of driving schools (118) • Managers at different levels, car manufacturers and policymakers (117) • Special groups (disabled, blind, etc.) (90)
Program content	• Education of traffic rules (152) • Traffic light principles (150) • Visibility (149) • Distraction (143) • Illegal overtaking and speed (137) • Effect of prescription and non-prescription drugs and alcohol on behavior of users (130) • Places of safe passage (129) • Benefits of safe passage (124) • Types of safe passage (124) • Mutual respect and lack of verbal and nonverbal aggression (123) • Possible risks while passing (123) • Observing longitudinal and transverse spacing (123) • Fatigue and drowsiness (121)	• Increased positive behaviors (121) • Technical educations (120) • Clean transport (119) • Demanding and acquaintance with citizenship rights (119) • First aid and medical emergencies (119) • Driving in certain weather conditions (117) • Seat belt (117) • Restraint (117) • Safety helmet (117) • Diseases and traffic behavior (117) • Conditional driving (113) • Right of priority (112) • Social and religious laws (traffic ethics) (111) • Sleep disorders (111)
Educational methods	• Audio and visual media (155) • Practical training (152) • Courses (144) • Modeling (138) • Campaign (127) • Simulators (122)	• Workshop (19) • Seminar (17) • Group discussion (16) • Social networks (15) • Material and spiritual fines (14) • Summary reports (13)
Instructors	• TV (163) • School and university teachers (153) • instructors of driver’s license	centers (147) • Parents (128) • Health worker (125) • Police (120)
Resources	• Manpower (29) • Sufficient budget allocation and financial administrative support (27) • Proper technology (19)	• Providing materials and physical space (18) • National Traffic Document (17) • Strong monitoring system (12) • NGOs and charities (11)
Evaluation	• Number of accident and deatches (17) • Observation of traffic behavior before and after educational program (16)	• Number of hospitalizations (7) • Air pollution (3) • Satisfaction of program (1)

### Target groups

The first theme of NRTSEP was target groups which consisted of 12 groups needing to receive TSEP that included, preschoolers to university students, youth, teachers, parents, elderly, motorcyclists, cyclists, pedestrians, drivers, driving license applicants, instructors and managers of driving schools, Special groups (disabled, blind, etc.), managers at different levels, car manufacturers and policymakers. More information on categories this theme, descriptions and sample of quotations can be found in Table 2.

**Table 2:** Target groups of the National Traffic Safety Educational Program

Target groups	Description	***Quotation***
Preschoolers to university students	Early childhood education was considered important in all discussions and interviews. Children should see safe behaviors in the cartoons and other programs in the early ages, because indirect education greatly affects their behaviors. On the other hands, a course of traffic must be included from kindergarten to university. According to the WHO’s definition, those below 18 fall into the category of children.	*“Behavior originates from childhood, becomes institutionalized, and grows.”* *“In elementary and secondary schools by age of children.”*
Youth	They are above 18 years old who might be students, soldiers, or involved in other activities. They should receive necessary education, since they are at the age of getting a driver’s license. On the other hand, post-school education should also continue.	*“Today’s university student is tomorrow’s manager, employee, or teacher.”* *“In barracks, dormitories, coffee houses,* etc.*”*
Teachers	This group is a role model for children and adolescents; therefore, they should learn a proper traffic behavior and transfer it to those who make the future. Moreover, teachers as those who are responsible for traffic safety education must receive specific training.	*“Teachers also have a special impact on students.”*
Parents	Due to the interaction between parents and children and the impact of their behaviors on each other, all participants and experts in this study expressed that mothers and fathers should be trained about safe traffic behaviors and observe these cases in the daily life.	*“Parents are the primary role model for children.”*
Elderly	The WHO classifies the elderly as vulnerable groups who are at a greater risk due to the reduced motor and cognitive skills.	*“He is 80 years old and driving at the speed of 180 km/h in a Pride.”type of car*
Motorcyclists	Motorcyclists are the major group at risk of injury and see the most severe damage in traffic accidents. It is essential that all motorcyclists receive the required trainings to reduce accidents as well as injuries during an accident [25].	*“Motorcyclists don’t care about the traffic lights”* *“He has become a motorcycle messenger down on the streets.”*
Cyclists	Cycling is a form of physical activity that improves human health. Moreover, if bikes are more used in the transport system, the air pollution will be reduced; but, the cycling schools need to be increased with professional and recreational training periods.	*“Safety cases are taught to everyone, including drivers and cyclists.”* *“There are a small number of cycling training and schools.”*
Pedestrians	In Iran, the rate of deaths caused by accidents among pedestrians in traffic accidents is above 30% [26].	*“The pedestrian doesn’t even know where to pass.”*
Drivers	Given the high rate of accidents, deaths, and injuries caused by driving in the country, the drivers need to receive special training programs.	*“Driving is not just about parallel parking.”* *“One is a driver while he has obtained his license 20 years ago.”*
Driving license applicants, instructors and managers of driving schools	Driver’s license centers give an identity of driver to the applicants, given that driving is not just about introduction to driving skills, the correct behaviors and rules related to traffic both as a driver and as a pedestrian must be taught to the applicants. For this purpose, the instructors and managers of driving schools must also receive special training to transfer it to students.	*“Even the instructor doesn’t care about the rules, what do you expect from the leaner?”* *“Driver’s license is not a right for everyone, it doesn’t mean that everybody is supposed to have it, it is a competence.”*
Special groups (disabled, blind, etc.)	The WHO classifies people with disabilities as vulnerable groups who require specific trainings.	*“He is passing through the crossroads in a wheelchair.”*
Managers at different levels, car manufacturers and policymakers	Based on ecological patterns of behavior change, variations at the scale of policymaking could affect other layers and levels, and its impact is often greater. If synchronized with other levels, it will have synergistic effects. Higher sensitivity to the traffic safety problem is required to make it a priority in all policymaking.	*“It must be sent to all policymakers and mangers.”* *“First, the managers must be trained.”*

In sum, we found that children below 18 years of age and students were very important target groups because they will be parents, managers, … in future.

### Program content

The second theme of NRTSEP was the content of program, which consisted of 27 educational contents that should be taught in different target groups. Table 3 shows the educational content of this program along with an explanations and quotations to select the relevant content.

**Table 3:** Content of the National Traffic Safety Educational Program

Program content	Description	Quotation
Education of traffic rules and regulations	Traffic laws and regulations for untrained users and limited educations for driver’s license applicants.	“Learn the laws correctly.” “Which cyclist, motorcyclist, or pedestrian have been trained so far?”
Seat belt	Use of a seat belt reduces injury and death.	“Seat belts are not used for rear-seat passengers.”
Child seat	This device is not used in Iran, especially for those over 5 years old.	“If a child seat is used, severe injuries turn into mild injuries”
Safety helmet	The most effective method is to reduce head injuries and number of mortality caused by motorcycle and bicycle accidents.	“Macan Band wore safety helmets in the concert while driving a motorbike. The day after, all youth were wearing a helmet.”
Benefits of safe passage	Benefits of safe passage both for pedestrians and two- or more-wheeled vehicle drivers must be taught.	“What is the benefit of the bridge? What is the line? Many think these are only decorations.”
Type of safe passage	Safe passage must be taught to all age groups of pedestrians, drivers and other road users. In many cases, the users are not aware of such things.	“Who has ever taught me how to cross the street” “The pedestrian is crossing diagonally through middle of the square.”
Place of safe passage	Place of safe passage should be taught to all road users.	“Pedestrians don’t know that they should not enter the highway. Drivers park in the pavement and pedestrians walk in the street”
Possible risks while passing	Problems that occur to all users while passing should be taught.	“Pedestrians will pass through the traffic lights and cars, and don’t care about the lines.”
Traffic light principles	–	“Have we (pedestrians) ever paid attention to the lights? Everyone thinks the lights are only for drivers.”
Observing longitudinal and transverse spacing	–	“Many people don’t know they have to drive between lines to avoid accidents.” “Not observing the distance causes chain accidents”
Overtaking and speed	–	“Instead of slowing down, the driver increases the speed on the pedestrian lines.” “For example, suspending a person’s license at unauthorized speed and having forced training courses”
Fatigue and drowsiness	–	“If we had sleep control training, the accidents would be declined after a year.”
Clean transport	Promoting public transport system and greater accessibility reduce the volume of traffic and pollution. Public transport infrastructures must be created and managed in accordance with world standards.	“In Tehran, 2015, a number of 5417 people died only due to air pollution. 85% of air pollution is caused by vehicles and motorcycles.
Demanding and acquaintance with citizenship rights	The aspect associated with introduction to traffic rights and needs. People must be able to demand self-identification needs in traffic environments, including road and vehicle.	“Naturally, the greatest demand impact is in the age group of 20–30.” “Demand associated with vehicle must be taught to people, so that people know about the features of a safe car that is the right of a citizen.”
Increased positive behaviors	Use of pavements for walking and not passing the roadway. Thanking the driver who allows passing the street and crossing over the right side and all the positive behaviors that can affect users in the traffic environment should be taught.	“We have a culture of courtesy that must be extended to driving.” “If I pass the pedestrian crossing, the next one also passes the line, so those who cross anywhere other than pedestrian crossing will be ashamed.”
Right of priority	–	“Here, everyone thinks they have the right to do anything. The driver says he has a right to cross first, the same goes for pedestrians.”
Mutual respect and lack of aggression with verbal and nonverbal movements	–	“I should respect the traffic light. This is for my safety. I should respect the officer and don’t argue with him.” “The passenger kicks the door of the taxi and insults the driver. The passenger slowly crosses the street to offend the driver.”
Social and religious laws (traffic ethics)	Every user should be taught about the rights of him/herself and others. In most countries around the world, any kind of traffic violation is the violation of public rights (Arasteh).	“Talking about social rights and teaching the accidental and traffic issues.” “Motorcycles shouldn’t come to the pavements. It is a violation of rights of others.”
Visibility	Discussing “visibility”, it is necessary to pay attention to the environmental structures and appropriate light for the passages. Also, it is about the society culture and dark clothes for women and elderly, and depending on clothes for education, the use of appropriate reflectors in the bags, shoes, buttons, etc. In this regard, the manufacturers must be used. In case of vehicles, it should be noticed that rear window and other parts as well as the headlights must be designed in a way to provide maximum visibility.	“It’s hard to see at nights. Some neighborhoods have no lights and we can’t see someone with a black cloak crossing the street. These accidents are very common in the streets. The passers die and the drivers get away.”
Distraction	Various distractions of eating, drinking, smoking, looking at environments, mobile phones, and talking with fellows should be taught to all road users.	“They should pay more attention. Both the driver and the passer while crossing the street should pay attention to both sides, but unfortunately,…” “Many accidents are caused by mobile phones.”
Technical educations	Technical knowledge needs of a person such as car safety conditions before moving, activities required at the time of tire burst, braking control, etc. Competence of a person for driving two- or more-wheeled vehicles and health of the person are the cases that require education.	“The technical aspects required for road and vehicles, so that a person knows about the slippery sign.”
Driving in certain weather conditions	Depending on various weather conditions in different seasons and also due to the specific climate conditions in different regions of the country, these cases are taught, training in mountainous, desert, and rainy areas.	“In the snow and frost, I can’t control the car because of tires.”
Effect of prescription and non-prescription drugs and alcohol on behavior of users	The use of alcohol, amphetamines, euphoric stimulants, psychotropic drugs, even authorized and prescription drugs, for treatment and their effects on users must be taught.	“They need to teach young people that driving under the influence can ruin the lives of others and destroy families” “Doctors must say the effect of drugs on driving while prescribing the drugs”
Conditional driving	Given the increased number of non-communicable diseases in the country, the case detection is required and a treatment must be considered for those with specific diseases. It is essential to consider certain rules for issuing conditional certification. The driver’s license of these people must be regularly checked for treatment. Diseases or disorders of individuals that affect their driving should be identified and managed through education and license revocation or limitation.	“No license will be eternal, and in case of non-competency, the license should be revoked.” “There was a case that after frequent accidents, the schizophrenia was confirmed, and when the police stopped him, he claimed to be a psychopath; this is interesting that his license was not revoked.”
Sleep disorders	One of the most important problems, especially in professional drivers, is sleep disorders, its symptoms and treatment methods. In overweight people, the neck circumference must be measured and sleep disorders should be evaluated.	“From talking about sleeping to other subjects.”
Diseases and traffic behavior	A variety of mental disorders such as hyperactivity, epilepsy, etc. Physical diseases such as cardiovascular diseases, hypertension, and personality problems should be screened and treated, and the necessary cases for management of these diseases during driving must be trained.	“Regarding the diabetes¸ the training must be done by the doctors. Some people always have chocolate in the car.”
First aid and medical emergencies	In case of an accident, what activities must be done to minimize damage? First aid, cardiopulmonary resuscitation, dealing with ordinary and injured people, transport of injured patient, movement of injured patient;	“Management in the event of an accident, reduced consequences, assistance, saving the life of others, and subsequent training, which are ignored, what could happen to someone who is injured after 3 months?”

Overall, it was found that Primary, secondary and tertiary levels of education is given to target group.

### Educational methods

One of the elicited themes in the current study was the educational methods, including 12 categories namely; audio and visual media, practical training, modeling, simulators, Courses, Campaign, workshop, seminar, group discussion, material and spiritual fines, social networks, summary and reports. Table 4 shows the Educational methods of NRTSEP along with descriptions and quotations for selecting relevant training practices.

**Table 4:** Educational methods of national traffic safety educational program

Educational methods	Description	Quotation
Audio and visual media	The most important Educational methods presented in the present study are IRIB documentary and educational films, slides and educational books, radio, educational banners and posters, pamphlets, leaflets, tracts, flip charts, and any channel that can reasonably pass the educational content to the users, so that a person can feel the main causes of the behavior.	“All tutorials should contain photos, documentary films, etc.” “Now, the main mission of television and radio is training of traffic behaviors. The required training materials for IRIB must be provided by Ministry of Health and the police.”
Practical training	Real and practical training of behaviors in real-world environments of society, such as traffic parks, streets, etc. that stabilizes behavior. For children and license applicants, the practical traffic safety training will be more effective. Regarding the first aid, the practical trainings will be more effective.	“After teaching, they better learn to cross the street with the police.”
Modeling	Different target groups have specific models and references that can be used as training practices, including movie stars, artists, teachers, parents, friends, and other important people can affect the behavior of users as role models.	“Considering important characters in TV series and movies as role models.” “Children consider parents, teacher, and television as role models.”
Simulators	Equipping driver’s license centers with simulators for driving training, training of pedestrians in different target groups with simulators, conditional driving, first aid, and effect of drug use on behavior with simulators might lead to reasonable behaviors in the real environment.	“If someone in the simulator sees what would happen in case of texting while crossing the street, it could happen in the real world.”
Courses	–	“Taking courses through health centers, schools and organizations involved in traffic ...”
Campaign	In traffic campaigns, a message is sent from all channels, such as TV, radio, municipality, police, etc. on a specific behavior that is one of the priorities, which influences people across the country.	“Many behaviors will be resolved using the campaign, such as campaign for the pedestrian crossing, child seat, etc.”
Workshop	Workshops for management courses for managers at different levels to increase the empowerment and literacy of managers.	“Traffic workshops for health system staff, ...”
Training seminars	Specialized seminars and meetings for experts on professional issues.	“In a more limited state such as seminar.”
Group discussion	Participation in group discussions creates a commitment to behavior, so this training method must be used depending on the target group and educational content.	“Having group discussions and talking about unhealthy behaviors.”
Social networks	Virtual and real networks in the community increase the communication between individuals. This increases the social support of individuals within the network. The use of social networks on training of traffic behaviors increases social support for individuals within the network and safe traffic behaviors.	“Using social media…” “People support in social networks…”
Material and spiritual fines	Severe and costly financial and moral penalties on the traffic violations are among the methods that make a person stop doing the violations, for which he/she has been punished. Preventive penalties, for example: Examining the driving records of a person prior to everything when applying for a job, especially jobs with high social aspects.	“Experience of costly fines is effective even more than culture and education. People have concluded that in case of any violation, 20% of salary must be paid as fines.” “Issuing a fine for one of the university professors for having traffic violations was sweeping in front of the university. This is the prevention.”
Summary reports	To educate policymakers and planners, the experts from different fields should be used, and the statistics on death, injury, loss of workforce, and side costs should be sent briefly to a table or chart.	“A brief must be sent to all different policymakers and managers.”

The majority of participants mentioned TV as the national media that can provide traffic education directly and indirectly for all the target groups. Other training methods must be used based on target group and educational content.

### Instructors

The fourth elicited themes in the current study was the instructor, including 6 categories, namely TV, university and school teachers, instructors of driver’s license centers, parents, health workers and police.

The most important instructor in the present study was TV as national media that can be successful in promoting traffic behaviors by broadcasting different programs to all target groups. TV can show the lost costs via subtitles by providing the costs and statistics related to blood money, death, and costs of traffic accidents and problems caused by high volumes of traffic, such as respiratory diseases, and provide specific training via special networks such as IRIB Salamat (Specified health broadcasting network)for survivors of accidents in the field of consequences, flashbacks, and psychological problems caused by accidents. It can also provide information about books published on traffic accidents and their prevention for different groups from various networks. TV also can show the phone number to report accidents and broadcast the experiences and trainings of successful countries in reducing traffic accidents. It can use the popular characters among the children and adults, such as Sia Saketi and Davood Khatar etc. (Well-known Iranian cartoon characters) In order to teach safe traffic behaviors and produce documentaries on the lives of accident victims and survivors. Also, a channel known as traffic channel can be established or a great deal of time can be dedicated to the traffic programs in the IRIB Salamat. According to an expert:

“*TV has a key role in traffic education*”.

Another category of instructor was school and university teachers that teach to target groups after being trained. A participant said:

“Students *accept more what the teacher says”.*

*.* An expert said: “*the general academic course on traffic health and safety must be educated through trained teachers in universities for all fields”.*

Instructors of driver’s license centers were another category of instructor theme. As one participant said:

*“ driving is not just about introduction to driving skills, the correct behaviors and rules related to traffic both as a driver and as a pedestrian must be taught to the applicants through driving schools*”.

Other educational trustees were trained parents. They can indirectly teach by their right behavior to children.

“*Mothers and fathers should be trained about safe traffic behaviors and observe these cases in the daily life”.*

The other major instructor was Iranian health network rural health workers (called Behvarzes). Traffic behavior, like other healthy behaviors, should be trained trough health workers but the Ministry of Health has neglected it. According to an expert:

“*Health centers as the accepted health promotion settings of people can have the important role in traffic behavior education”.*

Police were another instructor in NRTSEP. An expert said:

“*Police can train practical traffic behaviors in different settings such as schools, traffic parks, streets, etc. to establish behavior*”.

Because of the interaction between the target groups, if all 6 instructors mentioned above are able to educate properly, safety traffic behavior can promote.

### Educational resources

This theme highlighted educational resources containing seven categories as; manpower; sufficient budget allocation and financial administrative support; proper technology; Providing materials and physical space; National Traffic Document; Strong monitoring systems; NGOs and charities.

#### Manpower

One of the most important resources required for the program is manpower. According to experts,

“ *Human resources must be trained with different skills and abilities, because it is at the macro scale across the country*.”

#### Sufficient budget allocation and financial administrative support

The majority of the experts in the present study believed that a lead agency is essential as a way of budgeting and resource management. All organizations must pay a part of their budget for educational contribution. Therefore, the resources are integrated and collected. An organization responsible for traffic at the macro scale According to experts,

“*It must be determined who is responsible, the lead agency, traffic police, or the ministry of health? Each ministry should pay its own contribution. All the money should be collected and what has to be done with that money (integration and collection of resources), pooling, etc*.” Given that the loss of human resources in society causes a severe economic and psychological burden on society, the cost is more effective with larger costs for teaching safe behavior as a prevention level. Another Expert said,

“*According to Lougou, if you think that investment in field of education is expensive, you must calculate the costs of ignorance*.”

Another expert said,

“*President Rouhani (Iran president)declared in 2015 that we have 3.5% negative annual income balance and a mortality figure of 32000 people per year (17000 due to traffic accidents and the rest for air pollution caused by traffic), and GDP of 6.4%. Therefore, if we prevent the losses caused by accidents, not only 3.5% negative annual income balance is compensated for, but also we will have 3% growth*.”

#### Proper technology and providing materials and physical space

Most of experts expressed financial resources are used to provide the budget required for providing educational content with proper methods and multimedia, software programs, applications, electronic content for virtual education, physical space for training, equipment of cycling, motorcycling and driving schools, proper space and simulators, new tools and technologies, and traffic laboratories to create a safe behavior in universities and research centers, software and hardware technology for registry of accidents.

#### National Traffic Document

National traffic document was the important available resources that can be used for education of policy makers.

#### Strong monitoring system

A well-monitored audit structure is also necessary to spend the budget and expense considered in the program to build or modify the infrastructure. According to experts,

“*A strong monitoring must be taken into consideration as important resources to prevent abuse of costs for infrastructure in the program*.”

#### NGOs and charities

The advantages of donors, NGOs, insurance, and private companies can be used as program resources. Most of experts mentioned a lead agency can have the key role in resource organization for NRTSEP.

### Evaluation

The last theme of NRTSEP was evaluation, that consisted of five categories namely, Number of accidents and fatalities, Number of hospitalizations, Air pollution, Observation of traffic behavior before and after educational program and Satisfaction about the program.

## Discussion

The purpose of the present study was to develop a national educational program on traffic safety promotion. The importance of education is to the point that one of the pivots of the Decade of Action for Road Safety 2011–2020 is education [27]. One of the important indicators that reduce deaths caused by accidents in developed countries is paying attention to education [12]. In Iran, we have above 17,000 deaths caused by traffic accidents. It is predicted that for each death, 16 cases of accidents lead to hospital admissions and 400 cases require outpatient services [28]. The highest rate of admissions in hospital emergencies is caused by traffic accidents that imposes huge direct and indirect costs on the government and the public and accounts for a major share of annual budget of countries [29].

In order to reduce accidents, hospitalizations, and problems and also to reduce its costs, an educational program is required to promote the user safety behavior given the environmental, economic, political, cultural conditions along with changes in the road network and increased vehicle safety. Based on the results of the study, the national educational program on road traffic safety promotion was designed in 6 dimensions. The main dimensions of this program included, target group, educational program content, teaching method, instructors, resources, and evaluation method.

In the present study of the target group, included 12 groups of preschooler to university students, youth, teachers, parents, elderly, motorcyclists, cyclists, pedestrians, drivers, license applicants, instructors and managers of driving schools, special groups (disabled, pregnant women, blind, etc.), managers at different levels, car manufacturers, and policymakers. In other words, based on the results of the present study, various levels of society should have traffic safety education. Similar to the present study, the PRSE (Permanent Road Safety Education) is continued for learning traffic culture from childhood to the end of life at all levels of society in the successful countries of the world on lowering traffic accidents, such as the Netherlands [12]. Similar to the present study, children’s education has been considered because high-risk behaviors are more common in adults and children [30] Childhood, on the other hand, is a very important period for developing behavior [31]. The European Commission also identifies vulnerable groups such as the elderly, pedestrians, disabled people, and all road users as target groups that must be trained [32]. A review study is focused on education of vulnerable groups, such as children, the elderly, pedestrians, bicycles and motorcyclists in the Asia [33]. In Iran, due to the high number of traffic accidents and injuries, all target groups need to be trained. The emphasis of the people and the experts was on education from childhood until the end of life. Knowledge development is one of the pillars of road safety, which can affect drivers’ awareness and behavior on the road [13].

The content of the present program consisted of 27 items (Table 1), which is consistent with the educational content used in the world. In the present study, the education of the traffic rules and regulations and safe passage behavior (type of safe passage, place of safe passage, benefits and risks of safe passage) have been mentioned in the educational content for both pedestrians and other users. In Switzerland, the educational content consisted of two main sections: traffic safety, legal traffic behavior, compliance with traffic regulations, and environment [33] and have been successful in traffic education and reduce RTIs. Visibility, reflectors in clothing, bags and shoes, day lights, proper lighting for roads, post-accident care training, public transport to reduce traffic volumes, safe cycling and its infrastructures, education of managers at different levels, car manufacturers, and policymakers to improve vehicle safety, improvement of transport system, modification of road network structure, and greater vehicle safety, overtaking and speed, pedestrian safety, and place of safe passage, were the results of the present study that WHO has also pointed out [34]. Wearing seat belts, safety helmets, observing authorized driving speed, alcohol use, and first aid were the educational content obtained in the present study, Permanent education of theirs can reduce of RTIs [35–38].

In the present study, the educational methods included audio-visual media, practical education, role modeling, simulators, taking courses, campaigns, workshops, educational seminars, group discussions, social networks, financial and moral penalties, and brief reports, which is similar to the review study conducted by Jamaludin et al. [39]. The teaching methods in schools at Netherlands are done using traffic guides and distribution of books, pamphlets, films and CDs. In Germany, the traffic safety education is done via film and slides, websites, book distribution, simulators, games, practical street tours [12], and workshops [36]. The safety and traffic training course will be held in Iranian universities from 2018. This course was conducted as a national preliminary study in four stages. The results showed that before participating in the training courses, the level of knowledge of student traffic was low. Studies have also shown that “pedestrian safety” and “first aid” were the most useful and practical issues [40]. The educational method can varied based on target group and type of content. For example, in the driver license applicants, some behaviors such as the impact of using a mobile phone while driving can be simulated, so the person can somehow understand the reality. In the present study, educational instructors included TV programs, schools and university teachers, parents, teachers of driving schools, seminary, health workers, and police experts, and a part of which has been noted in a study conducted by Mark Lee and Al-Mansour [35]. Peden has also considered the health workers as educational authorities [36]. Mütze and Dobbeleer reported that in 81% of the states, the Ministry of Education is responsible for safety and traffic education, in collaboration with Minister of Transport, police, and government and nongovernmental agencies. In England and Wales, the local highway authorities are responsible for road safety education [37].

The participants of the present study considered IRIB and its programs as the most important educational authorities, which directly and indirectly affect the education and promotion of safe traffic behavior, and then, there are the teachers trained in schools who are responsible for education of students. In this study, the resources were human resources, appropriate technology, adequate budgeting and financial support, materials and physical space, national traffic document, strong monitoring system, and NGOs. In Turkey, Kenya and Egypt, NGOs have been involved in traffic safety promotion programs [36]. The necessary resources and training budget in the Netherlands are provided by the sustainable budget of traffic safety education. In Germany, the books and pamphlets for traffic courses are provided by the Traffic Education Association and are given to teachers for training. German educational resources are so rich that could have reduced the death rate of pedestrian children by almost 20 times. Since 1947, the legal authority of road safety has been given to local authorities in England, and by hiring road safety officers, they provide the education costs of the road safety program. The Swedish National Institute of Transport and Research, along with other transport and training institutions, develop educational programs and provide resources to the education target group [12].

Experts participating in the present study stated that the resources will be managed in case of a leader agency in the field of traffic in the country. This agency can monitor and evaluate the organizations’ function, lead the policymaking, and manage the resources, and therefore, the problem of inter sectional cooperation that wastes the resources will be resolved. In countries that have managed to reduce the number of injuries caused by traffic accidents, an agency or organization with sufficient authority is responsible for plans and activities to reduce traffic accidents and other organizations should have an interaction and collaboration with this agency [41, 42].

The results of the present study showed that the educational program should be evaluated. For evaluation of program, the items included the frequency of deaths caused by traffic accidents, number of accidents and hospitalizations before and after training program, frequency of safe and non-safe behaviors before and after training program, satisfaction with program, and air pollution. Some programs have evaluated traffic behaviors after educational programs [43]. Some of the programs have also focused on quality of program and subsequent behavior changes [37]. Some studies have investigated the mediating variables, such as knowledge, attitude, and safe behavior [44]. In the Netherlands, an annual evaluation is conducted by research institutes on the impact of traffic training on behavior of students and parents, and the headings and instructions are constantly being revised, varied, resolved, and modified. All educational products and sustainable education plans of road safety are also evaluated. In German schools, in case of traffic accidents for a student who is unfamiliar with traffic regulations, the relevant teacher is responsible. At the end of each year, the students are tested so both the student and teacher are evaluated [12].

The present designed program must be implemented, and during implementation at various levels, the level of learning, impact on society, and long-term investment must be evaluated.

### Strengths and weaknesses of the study

Currently, the educational programs are implemented in a dispersed and island manner for some groups, but these programs need to be implemented continuously to reduce the number of accidents, deaths and injuries. This is the first program that was conducted as a needs assessment of people in different groups and was validated by Iranian experts.

The weakness of the present study was that only residents of Tabriz were used in needs assessment and people from other parts of the country were not surveyed. The research team hopes that this program can be implemented, and changes in traffic behavior followed by a decrease in death rate can be seen.

## Conclusion

According to the people and professionals, Ministry of Education plays a key role in education and institutionalization of traffic behaviors, and the curriculum on traffic safety should be included in all grades and educational levels of students based on their age and are taught in a practical manner. Due to a high interaction between parents and children, the education of children will affect parents. Moreover, the television and radio should also provide the traffic education so that this interaction can continue to reach a safe traffic behavior. After the Ministry of Education and the media, the driver license centers are particularly important in education and promotion of safe behaviors. According to both groups of people and professionals, the education of managers at different levels and policymakers in establishing and modifying infrastructure and adopting and reforming laws are critical in the national educational program on traffic safety promotion. The experts particularly emphasized the need for a leader agency to manage the traffic accidents.

## Data Availability

The datasets generated and/or analyzed during the current study are not public available in order to protect the participants anonymit, but they are available from the corresponding author on reasonable request.

## Abbreviations

NRTSEP: National traffic safety educational program
RTI: Road traffic injuries
FGD: Focus group discussions
IRIB: Islamic Republic of Iran broadcasting
